# The Radcliffe Infirmary

**Published:** 1892-05-07

**Authors:** 


### HOSPITAL RECONSTRUCTION.

### THE RADCLIFFE INFIRMARY.

The quarterly general Court of the Governors of the Radcliffe Infirmary was held on Wednesday in the committee-room of the institution, under the presidency of the Treasurer, the Master of the University. We take the following report from the *Oxford Chronicle*:—

The Rev. C. J. H. Fletcher said he wished to call the attention of the Board to a scandalous attack on the infirmary which ought not, as it seemed to him, to pass unnoticed, and with respect to which he would conclude with a motion. Early in the present year Mr. Burdett, the editor of a medical paper called The Hospital, and also of the *Hospital Annual*, came to the infirmary to address their nurses about some pension scheme he was desirous of promoting. He entered the infirmary by the basement, and not unnaturally was impressed by its gloom. Though his stay was brief, and his inspection of the wards incomplete and superficial, there appeared soon afterwards in his paper an attack on the infirmary which combined the maximum of invective with the minimum of fact. His main point was that they should pull down the old front building and erect a new one in its place, but as they had already decided to remodel this old building, and devote it entirely to administrative purposes they did not take his advice. Their preference of their own judgment to his had brought on the infirmary another, and he thought a worse attack than the first. It was contained in one of the prefaces to the *Hospital Annual*, and he must read it to the Court. After praising the people of Derby for having raised £45,000 for a new infirmary, the Editor proceeded thus: "This prompt action of the good people of the county of Derby is in striking contrast to that of the county and city of Oxford. Oxford University has for centuries claimed to be the centre of culture and intelligence. Derby has claimed no such professions. Unfortunately, however, the condition of a portion of the Radcliffe Infirmary, Oxford, is so unsatisfactory as to compel the Governors to admit that a portion of the building ought to be pulled down and re-built. Notwithstanding this admission, and the fact that the whole of the necessary work may easily be done for an outlay of £15,000, this wealthy county, this illustrious University, has remained in a state of quiescence, and seems content to allow its hospital to remain inefficient, inadequate, and almost a bye-word among the hospitals of this country at the present time. How it has come to pass that the county of Derby should have immediately aroused itself to raise £50,000, whilst the county and University of Oxford is content to allow notorious defects to prevail in its hospital without an attempt to raise so small a sum as £15,000, we fail to understand. At the quarterly Court of the Governors, held in July, 1891, it was determined to make certain alterations and to build a new wing to the Radcliffe Infirmary, providing funds were forthcoming. The meeting was very thinly attended, however, and there seemed to be an absence of resolution and public spirit throughout the proceedings, as it was strongly urged that even £6,000, *mirabile dictu!* would be a very large sum to raise, and it would be better to try and get the money in driblets instead of going boldly forward with a strong case, and so to throw the responsibility upon the county, city, and University of Oxford. We hope that after the holidays wiser and more vigorous counsels will prevail, or all concerned must suffer a serious loss of prestige. How is it that the University of Cambridge, and the county of Cambridge, have brought their hospital to a high state of efficiency, while Oxford is content to leave its hospital measurably behind many institutions to be met with even in the smaller towns of England at the present time? This is an age in which humbug is recognised and nailed to the post. If, therefore, the University of Oxford and the county of Oxford is content to merit it, it will undoubtedly receive the contempt of the English nation for its inertness and want of public spirit, unless or until it awakens to the fact that the Radcliffe Infirmary at the present time is unworthy, inadequate, and altogether inefficient, having regard to the advance and progress which have taken place during the last twenty years, not only in sanitation, but in hospital administration all the world over." With regard to this piece of writing, which gave such strong expression to such hasty impressions, he thought there were three remarks to be made. In the first place, there could be no doubt that it was calculated to injure the reputation of the infirmary, and to diminish its income from subscriptions. The infirmary was absolutely without qualification, characterised as unworthy, inefficient, inadequate, almost a bye-word, and possessing notorious defects. In the second place, the mode of publication made the injury doubly injurious. The *Hospital Annual* was, he believed, accepted as an authority in the medical world. It was to be found in every hospital, and it was largely used by medical men, so that the libel was not like one which appeared in a paper and disappeared after the day of appearance. It remained on the table for one or two years. It was, in fact, a continuing libel, and in the third place, he thought they would all agree that this attack was grossly and absurdly mendacious.

The Rev. O. Ogle asked the date of the publication.

Mr. Fletcher said he could not tell exactly. It was for 1891-2. Sir Henry Acland brought this subject before the notice of the Committee of Management shortly after the publication of this book, and read what he had read to the Committee, and he moved that Sir Douglas Galton should be asked to associate with himself some other inspector to report upon the infirmary with special reference to this charge. An amendment was moved to that, that no notice should be taken of it, or that nothing should be done. Unfortunately, it was not perceived at the time that that was no amendment at all, but was simply a negative of the motion. The consequence was that all other amendments were shut out. One member of the staff was prepared with another amendment identical with the one he was going to move. It seemed to him that they were guardians of the reputation of the infirmary, and that they ought not to allow it to be thus gibbetted before the medical world. He thought also in the interest of all hospitals they ought to take some action in this matter. If these annual directories, instead of being what they supposed them to be, collections of facts and statistics, were to be made vehicles of false and calumnious charges against institutions, it would be a gross and cruel perversion of them, because a charitable institution was not in the same position as an individual or commercial company. A charitable institution had no legal remedy; and therefore the only course it seemed to him it could take was the one he proposed, which was "That the editor of the *Lancet* be requested to send an experienced inspector to report on the state of the infirmary with special reference to the attack made upon it in Burdett's current edition of the *Hospital Annual.*" He did not lay special stress upon this particular way of meeting the attack, but it was the best way that commended itself to his mind; but the point he wished to emphasise was that it would not be right for the Court to allow this serious charge against the Infirmary to go on without taking some notice of it.

Dr. Collier said as he was the member of the staff referred to by Mr. Fletcher, he should be prepared to second the motion. There was only one remark which he should like to make, and that was as to the comparison between Addenbrooke's Hospital at Cambridge, and the Radcliffe Infirmary. He knew Addenbrooke's Hospital very well, he had resided in it, and was a life governor, and he was quite certain of this, that the sanitary condition of the Radcliffe Infirmary was quite as good, if not better, than that of Addenbrooke's Hospital.

Mr. Porter: Do you propose that the enquiry should be an open one or by a committee?

Mr. Fletcher said it would be, he supposed, for the gentleman who came down here to decide how he would go to work. He had not looked so far forward as that. He thought that would be a matter for after consideration.

Dr. Collier said he took it that Mr. Fletchor wished to invite the editor of the *Lancet* to appoint a competent person to report on the sanitary condition of the infirmary.

Mr. Fletcher: To report on the condition of the hospital with especial reference to this attack.

Mr. Porter asked if it was to be limited, for he had heard many complaints about the infirmary, especially as to the food of the patients. Did they not think it would be much better to include every complaint?

Mr. Fletcher: I am not prepared to do that.

The Rev. O. Ogle remarked that Mr. Fletcher had said that there was a difference of opinion on the Committee, and he should like to know the numbers that were present, and to hear from somebody who supported the so-called amendment, that nothing should be done, a statement on the other side to Mr. Fletcher's, before they voted. He thought they should have the fullest information before such an important subject like this was voted upon. He had come there with a different opinion on the matter, not having heard Mr. Fletcher's statement, and he should like to ask the reasons which induced the majority of the Committee to determine that no notice should be taken of the attack. He should like also to ask whether Mr. Fletcher was correct in saying that a charitable corporation like that could take no legal steps in cases of acknowledged libel.

Mr. Fletcher said that from his knowledge of law a long time back his impression was, though he could not guarantee it, that he was correct.

The Secretary referred to the minutes, and said there were 21 members of the Committee present. Sir Henry Acland moved that in view of the attack made on the hospital contained in *Burdett's Hospital Annual*, 1891-92, Sir Douglas Galton be asked to join with himself some thoroughly efficient inspector and report on the state of the hospital, the report to be circulated among the Governors. To this an amendment was moved by Mr. Dewar that no notice be taken of Mr. Burdett's attack, which was carried.

The Chairman said as far as he recollected the ground of objection was that the attack was not worth answering, and that it would be a wise thing, under such circumstances of an apparently untrue onslaught being made upon them, that they should not go out of their way, but should leave it alone.

Mr. Symonds said he seconded the amendment, and he did so because he read in chapter 1 of *Burdett's Hospital Annual* the following: "This action on the part of a few notorious wirepullers has done this much of good, that it has called public attention to the unwarrantable assertions which some designing but ignorant persons are prepared to make against the best hospitals in the country." (Laughter.)

Mr. Ogle said he fancied at first this was a mere newspaper attack, but it was another matter when it was made in a book of this kind.

Mr. Winkfield said with regard to Derby Hospital that there was an elaborate system of ventilation running through the walls, which at various times had become connected with the sewers; an outbreak of illness occurred and it was found that nothing could be done but to pull down the whole building. That did not apply to the Radcliffe Infirmary.

The Chairman asked why the *Lancet* should be invited to undertake the enquiry, and what was to be done with the report if they got it? What was the real intention of the motion?

Mr. Fletcher said with regard to the *Lancet* he adopted that paper because Dr. Collier proposed it, and he knew more about the medical papers than he (the speaker) did. As to what they should do with the report he was not at all at a loss to say. They should publish it in all the medical papers and circulate it amongst their own Governors.

Dr. Collier remarked that he suggested the *Lancet* because they had undertaken many of these enquiries. Then if the report appeared in the *Lancet*, of course it would go exactly where the other report of Mr. Burdett's went, that was to all the big hospitals not only in England, but in other parts of the world.

The Chairman did not think referring the matter to the *Lancet* was so good as the course suggested by Sir Henry Acland. To make their hospital practically the battle-ground of party medical quarrels was exceedingly undesirable. He thought the wisest course was to do as they had already done, and that was to leave the matter alone. There was no proof that the report had in any way hurt them or was likely to. No one who knew the hospital believed it was unsanitary or that it was wholly inefficient. What the rest of England believed did not make much difference to them.

Mr. Daniel: The question is, has the situation altered since we came to a conclusion?

The Chairman: That was only the Committee.

Mr. Thornhill said the whole attack was extremely vague from beginning to end. It simply was that the hospital was not so good as this gentleman desired, and the citizens and members of the University were mean people not to take his advice. (Laughter, and Hear, hear.)

Mr. Fletcher, in reply, said it had been remarked that this attack was not calculated to do them any injury. That might be so far as Oxford was concerned, although he was not prepared to endorse that, but he was chiefly thinking of the medical world; and they must remember that they had to draw their officials, their Matron, and resident house physicians, not from Oxford, but from the medical world. It seemed to him if the idea was to be accepted that this hospital was a byword among the hospitals of the country—and people reading this book certainly would derive that impression—it was calculated to do them a great injury by depriving them of first-class officials. Of course if this attack was made by a person utterly unworthy of credit or of no reputation he agreed that it might do to pass it over, but Mr. Burdett was an authority; he had just published a great work on Hospitals; he was not a person they could pass over in that way.

Mr. Porter: What objection has the Court to enquiry? Don't you think that by passing it over you admit your guilt? (Laughter.)

The Chairman: Not the least in the world.

Mr. Thornhill: There is no guilt alleged.

Mr. Porter said he thought there was a danger of the outside public thinking that the Court did not care for enquiry, and that there was something in the statements. Outside Governors like himself did not know much of the infirmary, and if the enquiry did not do good it could not do any harm.

On a division four voted for the motion, and seven against.

Mr. Ogle asked if this vote precluded any notice being taken of the report.

The Chairman: Until the next Court.

